# Anatomic and functional leg-length inequality: A review and recommendation for clinical decision-making. Part II, the functional or unloaded leg-length asymmetry

**DOI:** 10.1186/1746-1340-13-12

**Published:** 2005-07-20

**Authors:** Gary A Knutson

**Affiliations:** 1 840 W. 17^th^, Suite 5, Bloomington, IN, 47404, USA

**Keywords:** Leg-length inequality, functional, low back pain

## Abstract

**Background:**

Part II of this review examines the functional "short leg" or unloaded leg length alignment asymmetry, including the relationship between an anatomic and functional leg-length inequality. Based on the reviewed evidence, an outline for clinical decision making regarding functional and anatomic leg-length inequality will be provided.

**Methods:**

Online databases: Medline, CINAHL and Mantis. Plus library searches for the time frame of 1970–2005 were done using the term "leg-length inequality".

**Results and Discussion:**

The evidence suggests that an unloaded leg-length asymmetry is a different phenomenon than an anatomic leg-length inequality, and may be due to suprapelvic muscle hypertonicity. Anatomic leg-length inequality and unloaded functional or leg-length alignment asymmetry may interact in a loaded (standing) posture, but not in an unloaded (prone/supine) posture.

**Conclusion:**

The unloaded, functional leg-length alignment asymmetry is a likely phenomenon, although more research regarding reliability of the measurement procedure and validity relative to spinal dysfunction is needed. Functional leg-length alignment asymmetry should be eliminated before any necessary treatment of anatomic LLI.

## Review

In Part I of this review, the literature regarding the prevalence, magnitude, effects and clinical significance of anatomic leg-length inequality (LLI) was examined. Using data on leg-length inequality obtained by accurate and reliable x-ray methods, the prevalence of anatomic inequality was found to be 90%; the mean was 5.2 mm (SD 4.1). The evidence suggested that, for most people, anatomic leg-length inequality is not clinically significant until the magnitude reaches ~20 mm (~3/4"). The phenomenon of the functional "short leg" will be considered in Part II of this review. The objective is to define functional "short leg", how it differs from anatomic LLI and explore any association with neuromuscular dysfunction. In addition we will review the apparent efficacy of heel lifts in some cases of mild anatomic LLI, plus muscular reactions to, and causes of, pelvic torsion.

## The functional short leg, or unloaded leg-length alignment asymmetry

The functional short leg, or unloaded leg-length alignment asymmetry (hereafter abbreviated as LLAA) is itself a phenomenon much discussed and little understood. Essentially, when a subject lies prone or supine, unloading the pelvis, the feet are examined, most often at the welt (heel-sole interface), for the presence of a "short leg" or alignment asymmetry. Some hold the opinion that anatomic LLI can be measured in this way [1]. The examination for unloaded leg-length alignment asymmetry as a sign of "neuromuscular dysfunction" is a clinical test commonly used by chiropractors [2,3]. Given the frequent use of this test as an indicator of a functional problem, it is important to know whether the unloaded leg check test is an indicator of an anatomic short leg, or whether the test is reliable and valid as an instrument to measure functional "short leg" and whether LLAA findings are contaminated by anatomic LLI.

Anatomic LLI is caused by a natural developmental asymmetry or a variety of other factors, including fracture, disease, and complications of hip replacement surgery. Given the long-term loading, the lumbopelvic structure may be expected to adapt via Heuter Volkmanns' law [4] and soft tissue changes [4,5], establishing the compensated structural changes as "normal". This adaptive response is seen in the change of lumbosacral facet angles noted by Giles [6]. A case study followed the effect of anatomic LLI caused by hip replacement surgery on subjective symptoms, unloaded LLAA checks and pelvic unleveling, reporting that adaptive changes occurred over a period of several months [7].

Using a device to measure standing pelvic crest unleveling, Petrone et al found excellent intra and inter-examiner reliability, and validity (ICC, 0.89–0.90) relative to anatomic leg length inequality determined by x-ray measurement in asymptomatic subjects [8]. However, the correlation between the pelvic level and femoral head heights was "substantially lower" in a low back pain group. This indicates that some sort of functional pelvic tilt or torsion was present in the low back pain population that was unrelated to their anatomic LLI. While the decreased correlation between pelvic tilt and LLI in the back pain group was not examined relative to a functional short leg, the connection between back pain and the biomechanically unusual pelvic torsion stands out.

Lumbar lateral flexion was studied in a group of subjects 10 years after LLI caused by femoral fracture that occurred *after *they were skeletally mature [9]. Despite the compensatory lumbar scoliosis, these subjects had symmetrical lumbar lateral flexion, prompting the authors to comment that the "...acquired leg-length discrepancy produced little permanent structural abnormality in the lumbar spine..." [9]. Significant anatomic LLI acquired after skeletal maturity does not result in adaptive structural changes within a 10-year period.

However, another study from the same orthopedic center looked at the effects of significant (mean 3 cm) LLI acquired *prior *to skeletal maturity [10] in now mature subjects (17–38 years old, mean 28). In this group, there was considerable asymmetry of lumbar lateral flexion after placing a lift under the short leg to level the pelvis. This indicates that the body had permanently compensated to the structural changes in the spine/pelvis.

**Figure 1:**
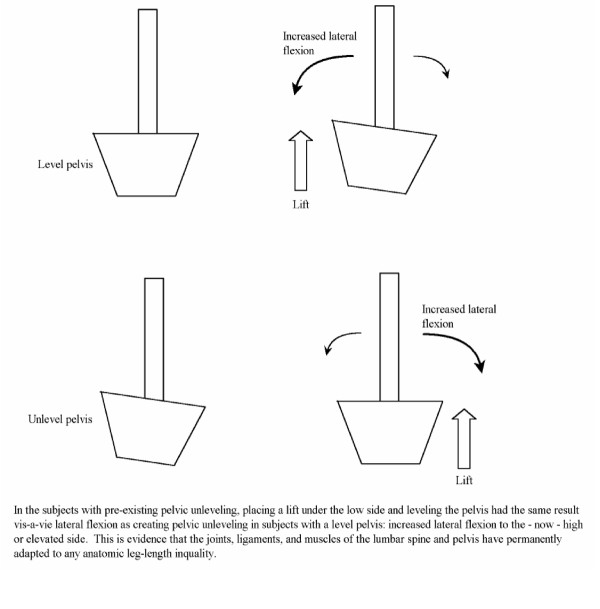
Effects of a lift in level and unlevel compensated pelvis.

This type of permanent compensation to pre skeletal maturity LLI was also found in subjects with pelvic unleveling. Young et al [11] found that placing a lift under the foot of a subject with no pelvic unleveling resulted in greater lumbar lateral flexion towards the now high iliac crest side. In subjects with pelvic unleveling, when the lift was put under the foot on the side of the low iliac crest in order to level the crest, lateral flexion was increased towards the formerly low crest side. If the body remodels and adapts to the pelvic unleveling/torsion caused by anatomic LLI, then by putting a lift under the side of the "low" iliac crest, one is actually raising what the body has adapted to as level. In other words, the unlevel pelvis of those with anatomic LLI has been adapted to and is now "normal", and putting a lift under the low side has the same effect as putting a lift under the leg of an even pelvis (Figure 1).

These two studies [10,11] provide evidence that in pre-skeletal maturity subjects, LLI and pelvic torsion – which describe the vast majority of LLI – adaptive changes take place in the muscles, ligaments, joints and bones to compensate for the imposed asymmetry. Because these adaptive compensations to the LLI have become anatomic, they are not likely to change as the body moves from a loaded (standing) to an unloaded (supine, prone) position. The nervous system also appears to compensate as demonstrated in the study by Murrell et al [12] in which there was no loss of stability in subjects with LLI, prompting them to point to "long-term adaptation by the neuromuscular system".

The persistence of pelvic torsion in subjects with anatomic LLI is supported by Klein [13] who found that such distortion remained in both standing and sitting positions. That pelvic torsion persists with the subjects' weight off the femoral heads indicates such torsion has been incorporated into the joints as the normal position. Rhodes et al demonstrated that the side and magnitude of prone and especially supine "short legs" were not significantly correlated with radiographic anatomic LLI, indicating they are separate phenomena [14].

The studies noted above provide indirect evidence that the pelvic torsion associated with childhood-onset anatomic leg-length inequality is adapted for and incorporated as normal. It follows then, that when an average person with an anatomic LLI and structurally compensatory pelvic torsion moves from a loaded (standing) to an unloaded (prone/supine) position, the torsion of the pelvis remains intact and the leg length at the feet/shoes would appear "even" on a visual check. The pelvis – joints, ligaments and muscles – have adapted to the anatomic LLI, making any torsion structural. It is this putative biomechanical adaptation that makes unloaded leg-length alignment asymmetry tests – the functional "short leg" tests – unreliable as a measure of anatomic LLI [14].

Unloaded LLAA is suspected to result from hypertonicity of suprapelvic muscles [15-17]. In a study of subjects with and without supine LLAA, Knutson & Owens found those with LLAA had significantly decreased endurance times for the erector (Biering-Sorensen test) and quadratus lumborum muscles [18]. Further, the side of LLAA significantly correlated with the side of the QL muscle quickest to fatigue. One of the causes of increased susceptibility of muscles to fatigue is hypertonicity. These results stand in contrast to Mincer et al [19] who suspected altered muscle fatigue profiles with anatomic leg-length inequality, but did not find such, providing further evidence that LLAA is a pathological process distinct from LLI.

When standing, the actions of the QL depend on whether the spine or the pelvis is stabilized. If the pelvis is stabilized, QL contraction laterally flexes and extends the spine [1,20,21]. With the spine stable, QL contraction pulls cephalically through its attachment to the posterior aspect of the hemipelvis [1,21]. This load on the posterior aspect of the iliac crest could act to rotate the ipsilateral anterior hemipelvis lower – an AS ilium – causing the pelvis to torque and having the opposite effect on the contralateral hemipelvis – a PI ilium. The degree of torsion (if any) would be dependent on the tension in the QL and the freedom of movement of the pelvis, and any pre-existing pelvic torsion due to anatomic LLI. However, if the subject now adopts an unloaded posture – supine or prone – QL hypertonicity is freed from the load of the body and able to lift the ipsilateral hemipelvis, hip and leg in the cephalic direction, producing leg-length alignment asymmetry at the feet. This model is in agreement with Travell and Simons who write, "In recumbancy, active TrPs [trigger points] shorten the [quadratus lumborum] muscle and can thus distort pelvic alignment, elevating the pelvis on the side of the tense muscle" [1].

## Clinical considerations

Now we can return to the dilemma of how lifts may have a positive effect on back pain and muscle activity given that most anatomic LLI is not clinically significant. Torsion of the pelvis as an adaptive structural compensation in anatomic LLI has been shown to be limited. If a person has pelvic torsion due to anatomic LLI near the limits of the body's ability to adapt, and QL hypertonicity with its ability to cause pelvic torsion is superimposed, muscular bracing reactions and pain could be the result. Indahl et al [22] found that stimulation of the sacroiliac joint capsule (in pigs) caused reflexive muscular responses, depending on what area of the joint (dorsal/ventral) was stimulated. They note that, "Irritation of low threshold nerve endings in the sacroiliac joint tissue may trigger a reflex activation of the gluteal and paraspinal muscles that become painful over time". Interestingly, stimulation of the ventral area of the SI joint produced reflexive contraction of the quadratus lumborum. It may be that a positive feedback loop could be established where QL hypertonicity leads to lumbar curvature and pelvic torsion which stimulates the SI joint leading to more QL hypertonicity, more lumbar curvature and pelvic torsion. It will be interesting to see if a similar muscular reflex to SI stimulation is found in humans.

Based on their research, Allum et al [23] proposed that rotation of the trunk excites joint receptors in the lumbar spine triggering muscular contractions – paraspinal muscles – for balance correction. While these receptors likely have adapted to any pelvic/lumbar rotation caused by anatomic LLI, further pelvic torsion caused by QL hypertonicity may stimulate the balance receptors causing reflexive muscular contraction. A lift would reduce the pelvic torsion and lower the proprioceptive balance triggers below threshold, eliminating chronic, painful muscular contraction.

In a case of additive effects of anatomic LLI and QL/suprapelvic hypertonicity on pelvic torsion, a lift used to level the pelvis would take the strain off the sacroiliac and associated joints and ligaments and decrease potentially painful muscular bracing. Thus, lifts can work to decrease back pain in people with what seem to be clinically insignificant amounts of anatomic leg-length inequality. Of course, it would be important for the clinician to explore reasons for any quadratus lumborum and other suprapelvic muscle hypertonicity and eliminate them to provide a complete correction. On the other hand, pure anatomic LLI in the range of and above 20 mm – the upward limit for adaptive compensation – may stimulate sacroiliac and/or lumbar proprioceptors causing reflexive and ultimately painful muscular contractions that will only be relieved by a lift to level the pelvis.

Reliable detection of LLI and LLAA is difficult, but not impossible. Research has shown the examination procedures for putative LLAA both prone [24] and supine [25] to have intra- and inter-examiner reliability. In a controlled setting, Cooperstein et al investigated the accuracy of a compressive prone leg check in subjects with proscribed amounts of artificial LLI [26]. They found the procedure to be highly accurate – able to detect a difference in leg length magnitudes as little as +/- 1.87 mm, and noted that, "...compressive leg checking would be expected to identify the short or shortened leg side, irrespective of magnitude, 95.4% of the time". In this authors opinion, while it is necessary to be able to detect a functional asymmetry above a baseline amount, the LLAA is more of a go/no-go test relative to a clinical decision. As such, accuracy in magnitude is not critically important past that lower limit amount. In other words, as an example, clinicians would only have to agree that an asymmetry above 1/8" exists, and not whether the asymmetry is 1/2" versus 3/16". Studies designed to examine intra- and inter-examiner reliability should keep this in mind.

In addition to reliability, the leg check procedure outlined by Cooperstein et al demonstrated concurrent validity as assessed against artificial LLI [26]. However, as noted by the authors, the clinical relevance of the procedure is unknown. Other studies in a clinical environment have demonstrated validity of the supine procedure by correlating LLAA to increased rated pain (VAS) intensity and recurrent back pain [27], lower SF-12 general health scores [28] and altered supra-pelvic muscle function [18].

Once any suprapelvic muscle hypertonicity has been relieved – and the causes may be multiple, including upper cervical joint dysfunction [18,29-33] – the effect of anatomic LLI can be investigated. This treatment sequence – removal of suprapelvic muscle hypertonicity causing LLAA prior to investigating anatomic LLI – is also recommended by others [1,34]. Patient history (activities that involve prolonged, repetitive loading) and symptomatic presentation should arouse suspicion regarding a clinically significant anatomic LLI. The most accurate method to determine anatomic LLI is the A-P lumbopelvic x-ray with the central ray at the height of the femoral heads. If x-ray is undesirable, tape measure from the ASIS to the medial malleolus, while unreliable for LLI in amounts less than 10 mm [35], may be accurate enough with larger asymmetries if the average of two determinations are calculated [36]. Using a succession of blocks of known thickness under the leg ipsilateral to the low iliac crest in order to level the pelvis also may aid in determining the amount of lift necessary [37,38]. Both of the non-radiographic methods are questionable regarding accuracy and reliability; however, anatomic LLI is not likely to become clinically significant at much less than 20 mm (~3/4"), and this level of asymmetry may be found with greater reliability. If anatomic LLI is determined to be clinically significant, a lift may be indicated. Danbert [39] reviews the proper application of lifts, should they be necessary.

## Conclusion

Anatomic leg-length inequality under 20 mm and leg-length alignment asymmetry caused by supra-pelvic muscle hypertonicity may interact in a loaded (standing) posture, but not in an unloaded (prone/supine) posture. Any leg-length alignment asymmetry due to suprapelvic muscular hypertonicity should be eliminated before any necessary treatment of anatomic leg-length inequality. By using this information, which is open to change based on new studies, the clinician may better understand the diverse and sometimes confusing findings relative to anatomic leg-length inequality and functional or unloaded leg-length alignment asymmetry, and be better able to make treatment recommendations.

## Competing interests

The author(s) declare that they have no competing interests.
